# Chemogenetic Modulation of Orexin Neurons Reverses Changes in Anxiety and Locomotor Activity in the A53T Mouse Model of Parkinson’s Disease

**DOI:** 10.3389/fnins.2019.00702

**Published:** 2019-07-30

**Authors:** Milos Stanojlovic, Jean Pierre Pallais, Catherine M. Kotz

**Affiliations:** ^1^Department of Integrative Biology and Physiology, University of Minnesota, Minneapolis, MN, United States; ^2^Minneapolis VA Health Care System, Geriatric Research, Education and Clinical Center, Minneapolis, MN, United States

**Keywords:** Parkinson’s disease, orexin, locomotion, anxiety, neuromodulation, DREADDs

## Abstract

Parkinson’s disease (PD) is the second most common neurodegenerative disease. PD symptomology is recognized as heterogeneous and in addition to motor function decline includes cognitive, mood, sleep, and metabolic disorders. Previous studies showed early reductions in anxiety and locomotion in the A53T mice model of PD. Since inflammation and astrogliosis are an integral part of PD pathology and impair proper neuronal function, we were keen to investigate if behavioral changes in A53T mice are accompanied by increased inflammation and astrogliosis in the hippocampus (Hipp) and motor cortex (mCtx) brain regions involved in the regulation of anxiety and locomotion, respectively. To test this, we used 3-, 5-, and 7-month-old A53T mice to examine anxiety-like behavior, locomotion, and expression of inflammation and astrogliosis markers in the Hipp and mCtx. Further, we examined the presence of alpha-synuclein accumulation in orexin neurons and orexin neuronal loss. The data show early reductions in anxiety-like behavior as well as increased locomotor activity, which was accompanied by inflammation and astrogliosis in the Hipp and mCtx. Due to the persistence of the orexin neuron population in A53T mice and the involvement of orexin in anxiety and locomotor regulation, we hypothesized that chemogenetic modulation of orexin neurons would reverse the observed reductions in anxiety-like behavior and the increases in locomotor activity in these animals. We showed that chemogenetic activation of orexin neurons in A53T mice restores anxiety-like behavior back to control levels without affecting locomotor activity, whereas the inhibition of orexin neurons reverses the elevated locomotor activity without any effects on anxiety-like behavior. This study exemplifies the complex role of orexin neurons in this model of PD and demonstrates the novel finding that changes in locomotor and anxiety-like behavior are accompanied by inflammation and astrogliosis. Together, these data suggest that the orexin system may play a significant role in early and late stages of PD.

## Introduction

Parkinson’s disease (PD) is a progressive neurodegenerative disease that affects approximately 2–3% of the elderly population (Poewe et al., 2017). It is defined as a neurodegenerative movement disorder distinguished by bradykinesia, resting tremor, stiffness, postural instability, and periods of freezing (Graham and Sidhu, 2010; Dickson, 2012). Importantly, PD is also associated with non-motor symptoms. Studies show that mood, cognition, and metabolic impairments may even precede the development of motor disorders (Goldman and Litvan, 2011; Tan, 2012; Davis and Racette, 2016; Anandhan et al., 2017), which is often considered the marker of disease onset. PD is characterized by the loss of dopamine neurons in the substantia nigra pars compacta and the formation of the proteinaceous fibrillar cytoplasmic inclusions called Lewy bodies. The main component of the Lewy bodies, the hallmark of PD, is alpha-synuclein (α-syn), a small acidic protein expressed in presynaptic terminals, particularly in the neocortex, hippocampus (Hipp), striatum, thalamus, and cerebellum (Iwai et al., 1995). The role of α-syn in PD pathology is well established, and mutations in α-syn genes are responsible for several forms of autosomal dominant PD (Polymeropoulos et al., 1997; Krüger et al., 1998; Singleton et al., 2003; Chartier-Harlin et al., 2004; Zarranz et al., 2004).

Hualpha-SynA53T (A53T) mice express the familial PD-associated A53T missense mutant form of human α-syn under the control of the murine prion promoter. Compared to other PD transgenic mouse models, A53T shows the complete α-syn pathology that is observed in humans (Dawson et al., 2010) and is extensively studied in the context of neurodegeneration, α-syn aggregation, and toxicity (Paumier et al., 2013). These mice spontaneously develop the neurodegenerative disease between 9 and 16 months of age with a progressive motoric dysfunction leading to death within 14–21 days of onset (Lee et al., 2002). Overexpression of A53T mutant human α-syn in A53T mice increases neuronal toxicity and impairs neuronal function (Xie et al., 2015; Lee et al., 2017). Inflammation and astrogliosis are contributing factors to PD (Phani et al., 2012), and A53T-related pathology (Gu et al., 2010; Fellner et al., 2011; Booth et al., 2017).

Orexin (hypocretin) is a neurotransmitter exclusively produced by orexin neurons located predominantly in the lateral hypothalamus (LH). These neurons show a surprisingly complex projection pattern (Sakurai et al., 2005; Yoshida et al., 2006) and were initially recognized to be integral to hypothalamic-regulated physiological functions such as eating behavior, sleep, and spontaneous physical activity (Kotz, 2006; Tsujino and Sakurai, 2009; Girault et al., 2012; Inutsuka and Yamanaka, 2013; De Lecea and Huerta, 2014; Perez-Leighton et al., 2016). Orexin is also involved in the regulation of other functions including mood, cognition, response to stress, anxiety, and pain (Johnson et al., 2012; Flores et al., 2014; Muschamp et al., 2014; Yeoh et al., 2014; James et al., 2017a; Mavanji et al., 2017; Razavi and Hosseinzadeh, 2017). Several studies implicate a noteworthy role of orexin in PD (Drouot et al., 2003; Fronczek et al., 2007; Thannickal et al., 2007; Baumann et al., 2008; Wienecke et al., 2012; Bridoux et al., 2013).

In this study we hypothesized that the chemogenetic modulation of orexin neuron activity will ameliorate A53T-associated changes in anxiety-like behavior and locomotor activity. To test this, we used the targeted expression of genetically modified designer receptors exclusively activated by designer drugs (DREADD) approach. A virus containing a DREADD construct encoded in an inverted open-reading frame and flanked by lox-p recombination sites was stereotaxically injected into the LH of orexin-Cre (orx-Cre) and orx-Cre/A53T mice. In orx-Cre mice, Cre-recombinase expression is driven by the Orx promoter, which is exclusive for orexin neurons. DREADD excitation using clozapine-*N*-oxide (CNO) restored anxiety-like behavior to control levels, whereas inhibition of orexin neurons reduced locomotor activity without affecting anxiety-like behavior. These results suggest that orexin neuron circuitry dysfunction in the locomotion and anxiety changes observed in A53T mice, a model of PD.

## Materials and Methods

### Animals and Ethics Statement

All experimental procedures in this study were approved by the University of Minnesota Animal Care and Use Committee. Mice were maintained on a 12 h light/dark cycle with chow and water *ad libitum*. Adult male C57BL/6J (WT), A53T, orx-Cre, and orx-Cre/A53T animals were used for this study. The orx-Cre mice were initially obtained from Prof. Takeshi Sakurai (Kanazawa University, Japan) and bred on the C57BL/6J background in our colony. Generation and initial phenotyping of heterozygous orx-Cre and wild-type mice was conducted, and has been described previously (Matsuki et al., 2009; Zink et al., 2018). The A53T mice were obtained from the Jackson Laboratory (Bar Harbor, ME, United States) and bred on C57BL/6J background in our colony. Heterozygous A53T mice were generated and characterized as described previously (Giasson et al., 2002). The orx-Cre/A53T mice were generated by crossing orx-Cre-positive females and A53T-positive males.

### Behavioral Test Battery

**FIGURE 1:**
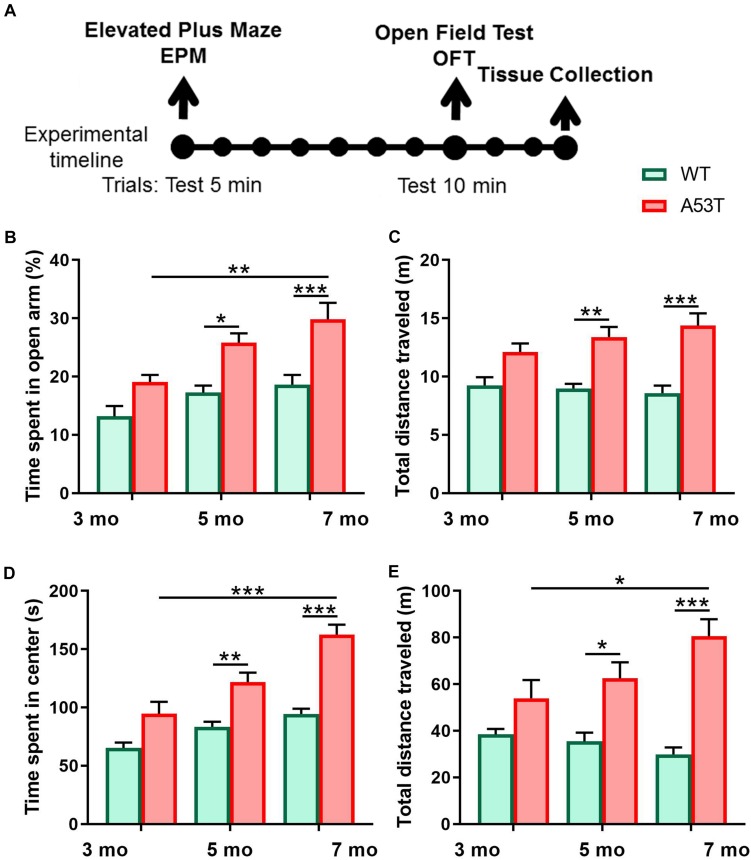
Anxiety-like behavior and locomotion and in 3, 5, and 7-month-old WT and A53T mice. **(A)** The timeline of the experimental procedure. Both WT and A53T animals (3, 5, and 7 months) were subjected to a behavioral test battery consisting of elevated plus maze (EPM) and open field test (OFT). Tests were conducted 7 days apart. Three days following OFT, animals were perfused and brains were collected. **(B)** The A53T mice spent more time in open arms of EPM compared to WT animals at 5 and 7 months of age. A progressive reduction of anxiety-like behavior was observed in the A53T mice. **(C)** The A53T animals covered more distance in the EPM at both 5 and 7 months of age. **(D)** Compared to age-matched controls, 5- and 7-month A53T mice spent more time in the center of the open field. **(E)** The A53T mice covered significantly more distance than WT animals at both 5 and 7 months of age. An aging-induced increase in locomotion was also observed in A53T mice (*n* = 10/group; ^*^p < 0.05, ^∗∗^*p* < 0.01, ^∗∗∗^*p* < 0.005).

The behavioral test battery consisted of two assays performed in the following order: elevated plus maze (EPM) and open field test (OFT) (Figure 1A). The EPM and OFT are two of the most widely used tests for measuring anxiety-like behavior and exploratory locomotion (Walf and Frye, 2007; Komada et al., 2008; Seibenhener and Wooten, 2015). These assays were conducted in an order that minimized the potential for behavioral effects from one assay to influence those in the next assay. The mice also had a 7-day rest time between tests to reduce carryover effects from prior tests and to enable CNO washout. The order of testing was the same for each mouse, and each mouse was tested only once per assay. All test trials were video-recorded, tracked, and analyzed with ANY-maze software (San Diego Instruments, San Diego, CA, United States). The maze or arena was cleaned thoroughly with 70% ethanol between each session. All tests in the battery were conducted by the same experimenter in the University of Minnesota Behavioral Core in the early light cycle phase between 8 am and 12 pm.

#### Elevated Plus Maze (EPM)

A Backlit EPM apparatus (Med Associates, Arlington, VA, United States) was used for this experiment. The maze was placed in the center of a room with its stage 95 cm above the floor level and all arms at least 70 cm away from any object in the room. The light intensity was set to 50 lux measured at the maze level. A video camera was installed 60 cm above the center of the maze. The video camera was connected to a computer and ANY-maze software (San Diego Instruments, San Diego, CA, United States) which was used to track and analyze the movement in real time. Animals were i.p. injected with 3 mg/kg of CNO dissolved in saline 30 min prior to the test. Subjects were placed in the center of the maze facing toward one of the open arms and allowed to freely explore the maze for 5 min. The total distance traveled, distance traveled in open and closed arms, as well as of time spent in closed and opened arms of the maze were recorded and analyzed. Percent of time spent in open arms (%) was calculated as (time in open arms × 100)/(total time).

#### Open Field Test (OFT)

An opaque, white acrylic arena (50 × 50 × 25 cm) was used for this experiment. A video camera was installed 40 cm above the center of the maze. The camera was connected to a computer and ANY-maze software (San Diego Instruments, San Diego, CA, United States) was used to track and analyze the movement in real-time mode. The light intensity was set to 250 lux measured at the arena level. Animals were i.p. injected with 3 mg/kg of CNO dissolved in saline 30 min prior to the test. Mice were placed in the middle of the arena and allowed to freely explore the arena for 10 min. Total distance traveled, distance traveled in center and edges of the arena, as well as time spent in center and edges of the arena were recorded and analyzed. Percent of time spent in the open area was calculated as (time in open area × 100)/(total time).

### Viral Injections and Drug Administration

Animals were anesthetized with isoflurane (1–4%) and placed in a stereotactic apparatus (Kopf Instruments). DREADD targeting was achieved by bilateral stereotaxic injection of a Cre-dependent AAV vector expressing a double-floxed inverted open-reading frame (DIO) around the DREADD transcript and a fluorescent tag (mCherry). Vectors (AddGene, Watertown, MA, United States) were injected into the LH (AP-1.8/DV-5.5/ML ±0.9 mm from bregma; 333 nl/5 min) (Franklin, 2008) of orx-Cre or orx-Cre/A53T mice. Control groups were injected with pAAV-hSyn-DIO-mCherry (AVV8, 2.1 × 10^13^ GC/ml) (cDREADD). Excitatory neuromodulation was achieved via Gq-coupled pAAV-hSyn-DIO-hM3D(Gq)-mCherry (AVV8, 2.5 × 10^13^ GC/ml) (qDREADD). Inhibitory neuromodulation was achieved via Gi-coupled pAAV-hSyn-DIO-hM4D(Gi)-mCherry (AVV8, 1.9 × 10^13^ GC/ml) (iDREADD). Animals recovered from the surgery for 2 weeks and were randomly assigned to appropriate experimental groups prior to testing.

### Immunohistochemistry

Mice were perfused intracardially with ice-cold saline, followed by 20 ml of 4% paraformaldehyde (PFA) in phosphate-buffered saline (PBS). Brains were harvested and post-fixed in 4% PFA/PBS overnight at 4°C, followed by 30% (w/v) sucrose in PBS solution at 4°C until the brains sank. The brains were imbedded in Optimal Cutting Temperature Compound (OCT; Sakura, Los Angeles, CA, United States), frozen in dry ice cooled ethanol, and then immediately cut. Forty micrometers of thick coronal brain sections was collected and stored in cryoprotectant [30% (w/v) sucrose, 30% (v/v) ethylene glycol, 1% (w/v) PVP-40 in PB]. Brain sections were washed six times for 5 min with 0.1 M PBS, pH 7.4. Antigen retrieval was performed using Antigen Unmasking Solution (Vector Laboratories, Burlingame, CA, United States). After initial washing (0.1 M PBS, pH 7.4, three times for 5 min) the sections were transferred to Antigen Unmasking Solution and incubated for 30 min at 90°C. The brain slices were then washed three times for 5 min in PBS and incubated with 5% normal horse serum in PBST for 2 h at room temperature. After washing three times in PBST, the sections were incubated with primary antibodies mouse alpha-synuclein (phospho S129) (anti-p-α-syn), Abcam, MA, United States; rabbit anti-glial fibrillary acidic protein (GFAP), Abcam, MA, United States; guinea pig anti-ionized calcium-binding adaptor molecule 1 (IBA1), Novus Biologicals, CO, United States; goat anti-orexin A, Santa Cruz, CA, United States; rabbit c-Fos, Santa Cruz, CA, United States; (1:1000) overnight at RT on a platform shaker. Brain sections were washed in PBST four times for 10 min after primary antibody incubation and incubated with secondary antibodies conjugated with Alexa Fluor dyes (donkey anti-mouse 555, donkey anti-rabbit 488, donkey anti-goat 647, donkey anti-guinea pig 647; 1:500, Invitrogen, Carlsbad, CA, United States). Brain sections were then washed four times for 10 min in PBST and then mounted with ProLong Gold mounting media (Invitrogen, Carlsbad, CA, United States).

### Immunofluorescence Imaging and Image Analysis

Immunofluorescence images for densitometry and IBA1 density experiments were captured using the Nikon Eclipse NI-E microscope (Nikon, Japan), with a monochrome Nikon Black and White camera DS-QiMc (Nikon, Japan). Each fluorochrome is represented as a pseudo-color in the images. For quantification of p-α-syn, GFAP, and IBA1, every 6th coronal sections from −1.22 to −2.30 bregma (Franklin, 2008) (four in total) for Hipp, and from 2.10 to 1.10 bregma (Franklin, 2008) (four in total) for motor cortex (mCtx) were collected, stained, and analyzed. Hipp and mCtx images were captured using 4× magnification. Optical density was determined with image analysis software (Image J, National Institutes of Health) by measuring the mean gray value of the Hipp. For IBA cell density, Z-stack images (5 μm step) were captured using 20× magnification. The IBA-positive cell densities in CA1 Hipp region and M1 mCtx region were determined using Image J by counting the positive cells in two areas of the CA1 and mCtx of every 6th section from 1.22 to −2.30 bregma (Franklin, 2008) (eight in total per structure) and divided by ROI area. For the p-α-syn localization study, every 6th LH section from −0.94 to −2.18 bregma (Franklin, 2008) (four in total) was stained and analyzed. To determine the percentage of orexin A-positive cells containing p-α-syn, every 6th coronal section from −0.94 to −2.18 bregma (Franklin, 2008) (five in total) was analyzed. Z-stack images (5 μm step) were captured using 10× and 40× magnification.

### Unbiased Stereology

Unbiased stereology analysis with optical fractionator probe within the Stereo Investigator 11.1.2 software (MBF Bioscience, Williston, VT, United States) was used to quantify the number of orexin A-positive cell population in LH. Sections were cut at 40 μm to allow for an 18 μm dissector height within each section after dehydration and mounting. Systematic sampling of every 3rd section was collected through the orexin field beginning at bregma −0.94 and finishing at −2.18 (Franklin, 2008), with the first sampled set of sections chosen at random. Sections were imaged using an Axio Imager M2 fluorescence microscope (Zeiss, Germany). Orexin field boundaries were used to outline contours at 5× magnification. Cells were counted using a randomly positioned grid system controlled by Stereo Investigator in a previously defined region in all optical planes. Guard zones were set at 10% of the section thickness to account for damage during the staining procedure. The grid size was set to 100 × 100 μm and the counting frame to 80 × 80 μm. Counting was performed on 63× magnification (oil). The average coefficient of error (CE, *m* = 1) ratio for all of the mice imaged was 0.085. Neurons were counted throughout the entire orexin field of each mouse to give an acceptable CE (Gunderson method) of 0.085 using the smoothness factor *m* = 1. The CE provides a means to estimate sampling precision, which is independent of natural biological variance. As the value approaches 0, the uncertainty in the estimate precision reduces. CE = 0.085 is deemed acceptable within the field of stereology. Cells were only counted if they touched the inclusion border or did not touch the exclusion border of the sampling grid.

### Statistical Analyses

All data were analyzed using either Prism 6.0 (GraphPad Software, San Diego, CA, United States) or SPSS (IBM, New York, NY, United States). Statistical analyses of phenotyping behavioral data were performed using a two-way ANOVA followed by Sidak’s *post hoc* analysis. Statistical analyses of DREADD behavioral data were performed using a one-way ANOVA followed by Tukey’s *post hoc* analysis. Densitometry and cell count data were analyzed using Student’s *T*-test.

### Experimental Design and Exclusion Criteria

The initial phenotyping study was performed in male 3-, 5-, and 7-month-old WT and A53T mice. The behavioral test battery consisting of EPM and OFT was performed. Tests were performed 7 days apart. Animal numbers used in EPM and OFT experiments: 3 months, *n* = 10/group; 5 months, *n* = 10/group; 7 months, *n* = 9/group. Three days following OFT, the animals were sacrificed, and their brains were collected for analysis (Figure 1A). Five-month-old mice used in the phenotyping study (*n* = 5 per group) were used for immunohistochemical (IHC) analysis. Every 6th coronal section containing LH −0.94 to −2.18 bregma and Hipp from bregma −1.22 to −2.30 were collected, stained, and analyzed. For the unbiased stereology study, every 3rd section from −0.94 to −2.18 bregma was collected and analyzed using the Stereo Investigator 11.1.2 software. Five-month-old WT and A53T animals were used for the unbiased stereology analysis (*n* = 4/group).

The DREADD study was performed in male 5-month orx-Cre and orx-Cre/A53T animals subjected to viral intracranial injections containing either cDREADD, qDREADD, or iDREADD. After a 2-week recovery period, animals were introduced to the behavioral test battery (EPM, OFT). Animal number used in EPM and OFT experiments: orx-Cre with cDREADD, *n* = 8; orx-Cre/A53T with cDREADD, *n* = 8; orx-Cre/A53T with qDREADD, *n* = 8; orx-Cre/A53T with iDREADD, *n* = 8. Tests were performed 7 days apart to prevent behavioral effects from testing in one assay to influence those in the next assay. Three days following the OFT, the animals were sacrificed, and their brains were collected for analysis (Figure 5A). All animals used in the DREADD study were perfused, and their brains were collected for injection placement confirmation. Coronal sections containing LH from −0.94 to −1.94 bregma were collected and analyzed. Animals were excluded from the experiment if *post hoc* histological analyses showed inaccurate viral injection placement. Mice were observed for neurological deficits and underperformance on behavioral tests, although none were observed. For DREADD expression and c-Fos analyses, qDREADD subjects were injected with either saline or CNO (5 mg/kg) 90 min prior to perfusion to confirm functional activation of the DREADD in orexin neurons by c-Fos (immediate early gene) labeling. Every 6th coronal section containing LH from −0.94 to −1.94 bregma (*n* = 5 per group) was stained for orexin A and c-Fos and then analyzed.

## Results

### Anxiety-Like Behavior and Exploratory Locomotion

To characterize anxiety-like behavior as well as exploratory locomotor activity we used 3-, 5-, and 7-month-old WT and A53T mice. Similar to previous studies, A53T mice showed a reduction in anxiety-like behavior (Graham and Sidhu, 2010; Oaks et al., 2013). A53T mice showed an increased time spent in the open arms of the EPM at 5 months of age (^*^p < 0.05; Figure 1B) and 7 months of age (^∗∗∗^*p* < 0.005; Figure 1B), and an increased time spent in the center area of the open field at 5 months of age (^∗∗^*p* < 0.01; Figure 1D) and 7 months of age (^∗∗∗^*p* < 0.005; Figure 1D). Progressive, aging induced reductions in time spent in the open arms of the EPM were observed in A53T mice (3-month A53T vs. 7-month A53T; ^∗∗^*p* < 0.01; Figure 1B). The time spent in the center area of the open field increased in A53T mice with age (3-month A53T vs. 7-month A53T; ^∗∗∗^*p* < 0.005; Figure 1D). Increased locomotor activity has been observed in previous studies (Unger et al., 2006; Graham and Sidhu, 2010; Oaks et al., 2013). A53T mice covered more distance in the EPM at 5 months of age (^∗∗^*p* < 0.01; Figure 1C) and at 7 months of age (^∗∗∗^*p* < 0.005; Figure 1C), as well as in the OFT at 5 months of age (^*^p < 0.05; Figure 1E) and at 7 months of age (^∗∗∗^*p* < 0.005; Figure 1E). Finally, A53T mice had aging-induced increases in the distance covered in OFT (3-month A53T vs. 7-month A53T; ^*^p < 0.05; Figure 1E).

### Expression of P-α-Syn, GFAP, and IBA1 in Hippocampus and Motor Cortex

**FIGURE 2:**
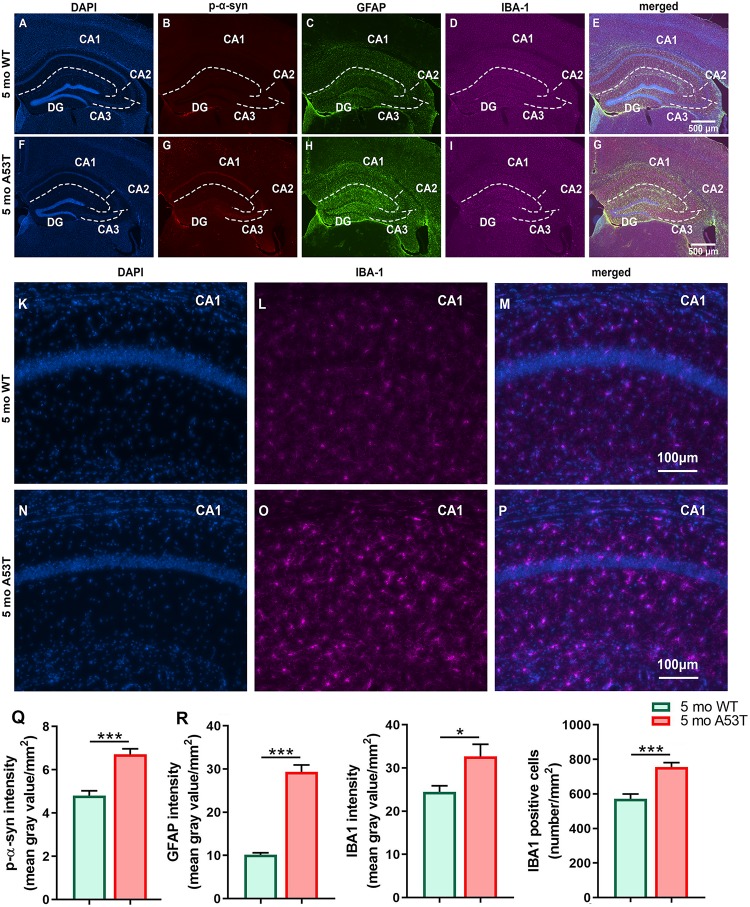
Immunofluorescence (IF) analysis of the hippocampal p-α-syn, GFAP, and IBA1 expression in 5-month-old WT and A53T mice. Representative IF microphotographs of the DAPI, p-α-syn, GFAP, IBA1, and merged image in 5-month WT mice **(A–E)** and A53T mice **(F–J)** used for densitometry analysis. Representative IF microphotographs of the DAPI, IBA1, and merged image in 5-month WT mice **(K–M)** and A53T mice **(N–P)** used for IBA1-positive cell density analysis. Image J was used to quantify the intensity of p-α-syn, GFAP, and IBA1 staining and density of IBA1-positive cells. The A53T mice showed an increased expression of the p-α-Syn **(Q)**, GFAP **(R)**, and IBA1 **(S)** compared to WT mice. **(T)** The A53T mice showed an increased density of IBA1-positive cells (*n* = 5/group; ^*^p < 0.05, ^∗∗∗^*p* < 0.005).

**FIGURE 3:**
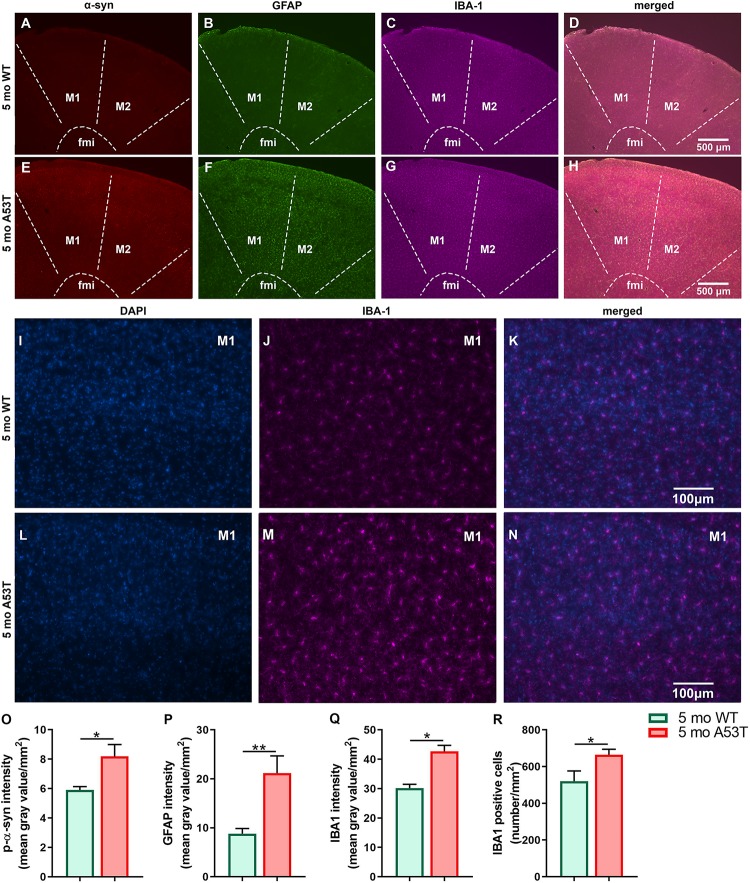
Expression of the p-α-syn, GFAP, and IBA1 in the motor cortex of 5-month-old WT and A53T mice. Representative IF microphotographs of the p-α-syn, GFAP, IBA1, and merged image in 5-month WT mice **(A–D)** and A53T mice **(E–H)** used for densitometry analysis. Representative IF microphotographs of the DAPI, IBA1, and merged image in 5-month WT mice **(I–K)** and A53T mice **(L–N)** used for IBA1-positive cell density analysis. Image J was used to quantify the intensity of p-α-syn, GFAP, and IBA1 staining and density of IBA1-positive cells. Increased expression of the p-α-Syn **(O)**, GFAP **(P)**, and IBA1 **(Q)** was observed in A53T mice compared to WT mice. **(R)** The A53T mice showed increased density of IBA1-positive cells (*n* = 5/group; ^*^p < 0.05, ^∗∗^*p* < 0.01).

It is thought that the overexpression of A53T mutant human α-syn in A53T mice increases neuronal toxicity and impairs neuronal function (Xie et al., 2015; Lee et al., 2017). PD-pathology induced changes in Hipp can be observed in representative microphotographs (Figures 2A–P). As expected, A53T mice showed an increased expression of p-α-syn (^∗∗∗^*p* < 0.005; Figure 2Q) in the Hipp. Inflammation and astrogliosis are present in PD (Phani et al., 2012), and in A53T-related pathology (Gu et al., 2010; Fellner et al., 2011; Booth et al., 2017). The expression of GFAP, a marker of astrogliosis, was increased in A53T mice in the Hipp compared to their age-matched controls (^∗∗∗^*p* < 0.005; Figure 2R). In the current study, an increase in IBA1 expression in the Hipp of the A53T mice was observed (^*^p < 0.05; Figure 2S). The increase in IBA1 expression was accompanied by increased numbers of IBA1-positive cells (^∗∗∗^*p* < 0.005; Figure 2T) in the CA1 region of the Hipp. Similar changes were observed in the mCtx (consisting of primary and secondary motor areas: M1, M2) as well (Figures 3A–N). A53T mice showed an increased expression of p-α-syn (^*^p < 0.05; Figure 3O). GFAP expression was increased in A53T mice (^∗∗^*p* < 0.01; Figure 3P). There was also increased IBA1 expression in the mCtx of the A53T mice when compared to age matched controls (^*^p < 0.05; Figure 3Q). Finally, an increased density of IBA1-positive cells (^*^p < 0.005; Figure 3R) was observed in mCtx.

### The Number of Orexin Neurons in Lateral Hypothalamus and Hippocampal P-α-Syn Expression

**FIGURE 4:**
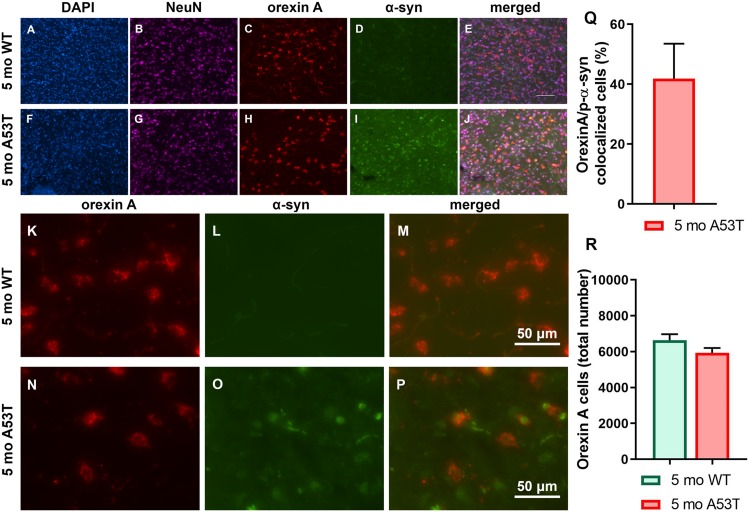
Immunofluorescence analysis of p-α-syn expression in orexin neurons and number of orexin neurons. **(A–J)** Representative IF microphotographs of the DAPI, NeuN, orexin A, p-α-syn, and merged image in 5-month WT mice **(A–E)** and A53T mice **(F–J)** showing a lack of the p-α-syn expression in 5-month WT mice and the presence of the p-α-syn in the orexin A-positive cells of the 5-month A53T mice. **(K–P)** Representative IF microphotographs of the orexin A, p-α-syn, and merged image in 5-month WT mice **(K–M)** and A53T mice **(N–P)** showing the presence of the p-α-syn in the orexin neurons of the 5-month A53T mice. **(Q)** Percentage of orexin neurons expressing p-α-syn defined as orexin A/p-α-syn colocalized cells in 5-month A53T mice. **(R)** Unbiased stereology analysis showed that there is no difference in the number of the orexin A-positive neuron between 5-month WT and 5-month A53T mice (*n* = 5/group).

As mentioned above, α-syn is associated with neuronal function impairment and even death (Xie et al., 2015; Lee et al., 2017). Furthermore, there are strong indications that orexin function is impaired in PD (Baumann et al., 2008; Takahashi et al., 2015) in addition to the loss of orexin neurons being present in the late stages of PD (Fronczek et al., 2007; Thannickal et al., 2007). Interestingly, p-α-syn aggregations were observed in orexin neurons (Figures 4A–P). Although 41.87 ± 11.66 (mean ± SEM; Figure 4Q) of the orexin neurons contained p-α-syn aggregations, it did not affect the number of the orexin neurons in the LH (Figure 4R), indicating an absence of orexin neuron loss in A53T mice at 5 months of age.

### Chemogenetic Manipulation of the Orexin Neurons Activity in the A53T Mice

**FIGURE 5:**
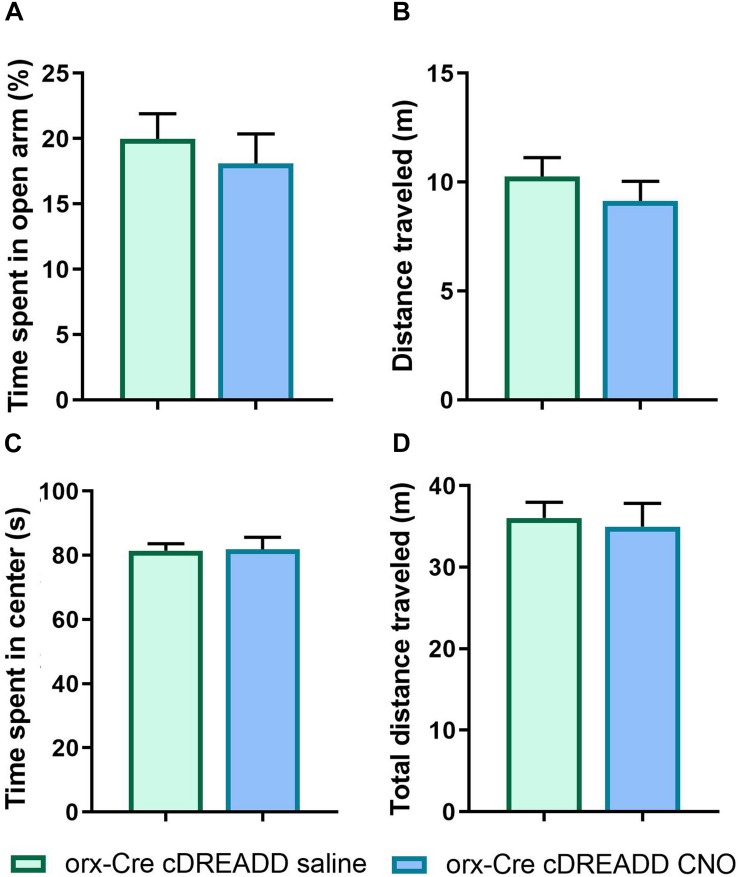
CNO effects on anxiety-like behavior and locomotion in 5-month orx-Cre male mice. Young 5-month mice received intracranial injections of control DREADD virus (cDREADD, pAAV-hSyn-DIO-mCherry). **(A,B)** CNO treatment did not affect distance traveled or time spent in open arms in EPM. **(C,D)** The time spent in open arms was not affected by CNO treatment as well as time spent in center of the OFT arena (*n* = 7/group).

**FIGURE 6:**
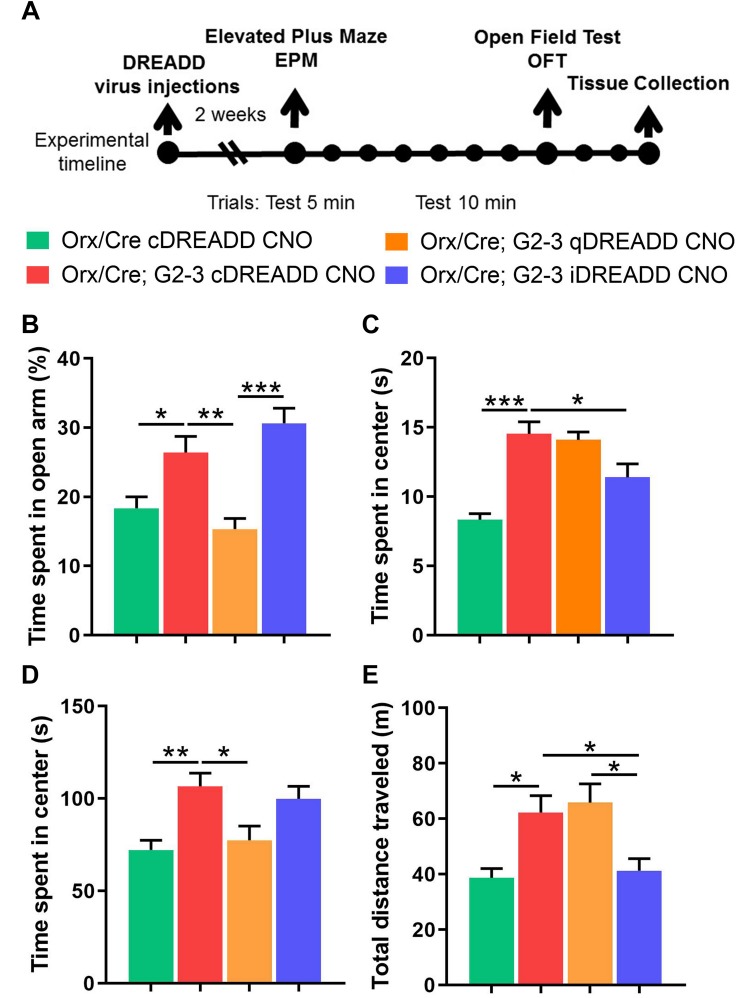
Chemogenetic modulation of anxiety-like behavior and locomotion in 5-month-old A53T mice. **(A)** The timeline of the experimental procedures. The orx-Cre and orx-Cre/A53T mice received intracranial viral injections. The orx-Cre mice received virus containing control DREADD construct, while orx-Cre/A53T mice received virus containing either control DREADD, inhibitory DREADD, or excitatory DREADD constructs. After 2 weeks of recovery time, a behavioral test battery (EPM and OFT) was performed. Tests were conducted 7 days apart. Three days following the OFT, animals were perfused and brains were collected. **(B)** Compared to control mice (orx-Cre), orx-Cre/A53T mice spent more time in open arms in the EPM. Activation of the orexin neurons by qDREADD reduced the time spent in the open arms of the EPM in orx-Cre/A53T mice while iDREADD-induced inhibition did not have a significant effect on the orx-Cre/A53T mice. **(C)** The A53T mice covered more distance in the EPM compared to control mice. Activation of orexin neurons by qDREADD failed to further increase the locomotion in orx-Cre/A53T mice. However, iDREADD-induced inhibition reduced the distance covered, restoring the level to that of control mice. **(D)** The orx-Cre/A53T mice spent less time in the center area of the open field compared to that of the control group. Activation of orexin neurons by qDREADD reduced the time spent in the center to levels observed in control animals. Inhibition of orexin neurons in the orx-Cre/A53T mice by iDREADD did not affect the time spent in the center of the open field. **(E)** The orx-Cre/A53T mice covered more distance in the OFT compared to that of the control, orx-Cre mice. Activation of the orexin neurons by qDREADD did not affect the distance covered by A53T mice, while chemogenetic inhibition of orexin neurons (iDREADD) reduced the locomotion relative to that in orx-Cre/A53T mice given the control cDREADD and those given the excitatory qDREADD. The DREADD-induced inhibition of the orexin neurons completely restored locomotor activity to that of the control, orx-Cre mice (*n* = 8/group; ^*^p < 0.05, ^∗∗^*p* < 0.01, ^∗∗∗^*p* < 0.005).

Prior to pursuing chemogenetic studies, we addressed a recent report (Gomez et al., 2017) indicating that CNO does not readily cross the blood–brain-barrier *in vivo.* Further, it was reported that CNO converts to clozapine *in vivo*, which has antipsychotic properties and may affect locomotor activity. Therefore, to exclude the possible independent actions of clozapine as a possible confound in our assay readouts, prior to the experiment described in Figure 6, we performed EPM and OFT assays in orx-Cre cDREADD (control) mice to assess if CNO alone affected anxiety-like- and locomotor behavior. As shown in Figures 5A–D, there were no effects of CNO on either of these endpoints, suggesting that the conversion of CNO to clozapine does not affect the outcomes.

Orexin is involved in the regulation of complex emotional responses. Studies show that orexin is a strong modulator of depression and anxiety states (Johnson et al., 2010, 2012; Lungwitz et al., 2012), and locomotor activity (Burgess, 2010; Kosse et al., 2017). To test if chemogenetic orexin neuronal activation modulation can mitigate changes in anxiety-like behavior and locomotion in A53T mice, we used a behavioral test battery consisting of EPM and OFT (Figure 6A). Compared to the control (orx-Cre) animals, orx-Cre/A53T mice spent more time in the open arms of the EPM (orx-Cre cDREADD CNO vs. orx-Cre/A53T cDREADD CNO; ^*^p < 0.05) (Figure 6B). DREADD-induced activation of orexin neurons reduced the time spent in the open arms of EPM (orx-Cre/A53T cDREADD CNO vs. orx-Cre/A53T qDREADD CNO; ^∗∗^*p* < 0.01) (Figure 6B). DREADD-induced inhibition of orexin neurons did not affect the time spent in open arms of the EPM; however, a difference was observed between the findings from animals treated with the excitatory and inhibitory DREADDs (orx-Cre/A53T qDREADD CNO vs. orx-Cre/A53T iDREADD CNO; ^∗∗∗^*p* < 0.005) (Figure 6B). A53T mice covered more distance compared to control mice (orx-Cre cDREADD CNO vs. orx-Cre/A53T cDREADD CNO; ^∗∗∗^*p* < 0.005) (Figure 6C) in the EPM. Chemogenetic inhibition of orexin neurons induced a reduction in general locomotion in A53T mice in the EPM (orx-Cre/A53T cDREADD CNO vs. orx-Cre/A53T iDREADD CNO; ^∗∗∗^*p* < 0.005) (Figure 6C). In the OFT, the A53T mice spent more time in the center of the arena (orx-Cre cDREADD CNO vs. orx-Cre/A53T cDREADD CNO; ^∗∗^*p* < 0.01) (Figure 6D). DREADD-induced activation of orexin neurons reduced the time that mice spent in the center of the open field (orx-Cre/A53T cDREADD CNO vs. orx-Cre/A53T qDREADD CNO; ^*^p < 0.05) while inhibition did not have any effect on the A53T mice (Figure 6D). The A53T mice covered more distance compared to control mice (orx-Cre cDREADD CNO vs. orx-Cre/A53T cDREADD CNO; ^*^p < 0.05) (Figure 6E) in the OFT. Activation of orexin neurons did not affect the covered distance in A53T mice, while DREADD-induced inhibition reduced the distance covered (orx-Cre/A53T cDREADD CNO vs. orx-Cre/A53T iDREADD CNO; ^*^p < 0.05) (Figure 6E). Finally, there was a significant difference in distance covered between A53T mice given the excitatory DREADD and those given the inhibitory DREADD (orx-Cre/A53T qDREADD CNO vs. orx-Cre/A53T iDREADD CNO; ^*^p < 0.05) (Figure 6E).

### Confirmation of Injection Placement and DREADD Functionality

**FIGURE 7:**
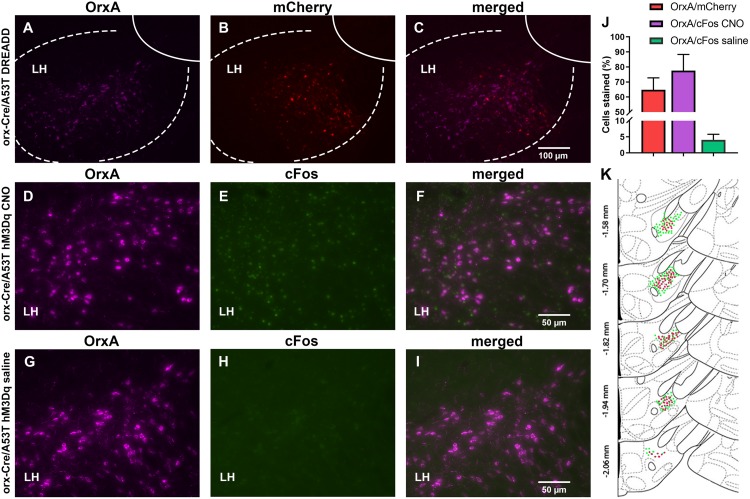
DREADD expression and functionality confirmation. **(A–C)** Representative images displaying viral expression of DREADDs in the LH. Orexin A (OrxA)-positive neurons in purple **(A)**, mCherry-positive neurons in red **(B)**, and merged images **(C)**. **(D–I)** Representative images displaying c-Fos (early gene) expression. The LH of excitatory DEADD animals was treated with CNO 90 min prior to perfusion **(D–F)** and excitatory DEADD animals were treated with saline 90 min prior to perfusion **(G–I)**. Orexin A is shown in purple **(D,G)**, c-Fos in green **(E,H)**, and then the merged images **(F,I)**. **(J)** The number of OrxA/mCherry and OrxA/c-Fos colocalized cells. **(K)** Schematic drawings displaying the spread of viral expression along the LH; green OrxA expressing cells, red mCherry expressing cells (*n* = 5/group).

The orx-Cre/A53T mice used in the DREADD study received bilateral DREADD viral injections. IHC analyses confirmed the selective expression of hM3Dq-mCherry in orexin neurons (Figures 7A–C,K). Clear co-localization of orexin A and hM3Dq-mCherry-positive cells was observed in cDREADD, qDREADD, and iDREADD mice (OrxA/mCherry total mean ± SEM, 64.75 ± 7.98; Figure 7J). Higher magnification images were used to estimate orexin neuronal-specific expression of the immediate early gene, c-Fos (Figures 7D–I). Measurement of c-Fos expression after CNO administration indicated that a majority of orexin neurons responded to CNO (OrxA/c-Fos CNO mean ± SEM, 77.53 ± 10.80) (Figure 7J). The group of animals that received saline had minimal co-expression of orexin and c-Fos (OrxA/c-Fos saline mean ± SEM, 4.08 ± 1.70; Figure 7J).

## Discussion

Parkinson’s disease is now recognized as a heterogeneous, complex disease that encompasses motor, cognitive, mood, and metabolic disorders (Goldman and Litvan, 2011; Tan, 2012; Anandhan et al., 2017). The pathology involves different brain regions, neurotransmitter systems, signaling pathways, and protein aggregations. Orexin neurons are thought to be involved in PD pathology, based on impairments in arousal and sleep in PD and the major role of orexin neurons in these functions (Abbott et al., 2005; Dhawan et al., 2006; Baumann et al., 2008; Asai et al., 2009; Compta et al., 2009).

The first goal of this study was to identify possible early changes in anxiety-like behavior and general locomotion in A53T mice and if these changes were accompanied by changes in orexin neurons, Hipp, and mCtx-related pathology. To address this, we used 3-, 5-, and 7-month-old WT and A53T mice and performed EPM and OFT behavioral assays. Our results are consistent with previous studies demonstrating age-related progressive reductions in anxiety-like behavior and increases in locomotor activity (Graham and Sidhu, 2010; Paumier et al., 2013) in A53T mice. As part of the limbic system, the Hipp is heavily involved in the regulation of anxiety (Walf and Frye, 2007; Xiang et al., 2011). Furthermore, a recent study showed the existence of “anxiety” cells in the hippocampal–hypothalamic circuit (Jimenez et al., 2018), suggesting that hypothalamus–hippocampal communication is essential for anxiety regulation. The roles of the mCtx and the orexin system in locomotion are well established (Zeitzer et al., 2004; Burgess, 2010; Kosse et al., 2017; Zhang et al., 2017; Heindorf et al., 2018). Today it is known that mood disorders, psychosis, and cognitive changes are all extremely common in PD. Yet several ideas need to be considered before interpreting data from this study: first, this study addresses very early stages of the disease. Early, pre-clinical stage symptoms of PD are an understudied area (Anderson, 2004; Siderowf and Lang, 2012), and thus whether behavioral symptoms occur in a linear fashion over time or whether they have a non-linear trajectory is unknown. Secondly, this animal model, particularly at this stage, does not mimic DOPA system impairment, and loss of the DOPA neurons in the ventral tegmental area, the origin of mesolimbic dopaminergic projection, is the most likely neuropathological cause of mood symptoms in PD (Anderson, 2004). Rather, this model is based on α-syn-induced pathology (Nakamura, 2013), and interpretation of the data needs to be taken within that context. Thirdly, it is well known that one of the dominant and earliest traits in PD patients are disinhibition and increased impulsivity, both of which are strong modulators of the observed behavior (Antonelli et al., 2011; Leeman and Potenza, 2011).

In the current study, we observed the presence of p-α-syn, increased expression of GFAP and IBA1, as well as an increase in IBA1 cell count in the Hipp and mCtx of 5-month-old A53T mice. Accumulation of p-α-syn, inflammation, and astrogliosis are considered factors that can impair Hipp and mCtx function. This is in agreement with earlier studies showing PD-related pathology present in Hipp (Norris et al., 2007; Paumier et al., 2013; Teravskis et al., 2018) and mCtx (Giasson et al., 2002; Martin et al., 2006) as well as impairment of Hipp and mCtx function in A53T mice. Further, we observed p-α-syn accumulation in orexin neurons, and the present data suggest possible impairments in orexin neuronal function. Given the presence of neurodegeneration of orexin neurons in PD (Fronczek et al., 2007; Thannickal et al., 2007), one of the main concerns in this study was whether enough orexin circuitry is preserved to retain significant neuromodulation capacity. To assess if there is orexin neurodegeneration present in A53T mice, we performed unbiased stereology analysis. This analysis showed no change in the number of orexin neurons in the 5-month WT vs. 5-month A53T mice. Given the pre-disease onset age group being studied, these results are not surprising and do not exclude the possibility that neurodegeneration of orexin neurons occurs in the later stages of the disease.

To determine if *in vivo* modulation of orexin neuron activity could mitigate A53T-related behavioral changes, we used the chemogenetic (DREADD) approach. First, to address concerns related to possible off-target effects of the designer ligand (CNO; Gomez et al., 2017) we performed EPM and OFT assays in orx-Cre cDREADD (control) mice. These studies confirmed that CNO treatment (3 mg/kg) did not have effects in control mice (Figure 5), mitigating concern over off-target and independent effects of clozapine. Mice prepared with either control, stimulatory, or inhibitory DREADD constructs were subjected to the OPM and EFT behavioral assays. The data show that targeted activation of orexin neurons restored anxiety-like behavior to levels observed in the control mice, while inhibition of orexin neurons did not affect anxiety-like behavior (Figure 6). The observed reductions in anxiety-like behavior in the A53T mice may be related to the imbalance in both excitatory and inhibitory neurotransmission in the Hipp (Simon and Gorman, 2006; Nuss, 2015). The orexin system is capable of modulating Hipp function in several ways. First, direct projections from orexin neurons to the different fields in the Hipp can increase excitation through G-protein-coupled orexin receptors (Sakurai et al., 2005; Yoshida et al., 2006; Farahimanesh et al., 2018). Secondly, orexin neurons can increase the release of gamma-aminobutyric acid (GABA) and glutamate in the Hipp through the medial septum (Sil’kis, 2013). Orexin directly affects components of the reinforcement circuit, such as neuronal populations in the prefrontal cortex, ventral striatum, amygdala, and ventral tegmental area. The Hipp is another component of this neuronal circuit and its activity can be modulated by upstream components of the circuitry (James et al., 2017b). Finally, orexin regulates mesolimbic dopamine signaling (Calipari and España, 2012), which is particularly interesting for PD research given the predominance of dopamine system impairment in this disease (Isaias et al., 2016; Surmeier, 2018). It is possible that the activation of orexin neurons consolidates the impaired Hipp function through any of the above-mentioned means, which in return restores anxiety-like behavior levels in A53T mice to normal.

The orexin system plays a major role in the regulation of physical activity (Zeitzer et al., 2004; Burgess, 2010). In our recent study (Stanojlovic et al., 2019), we observed that DREADD-induced activation of orexin neurons increased locomotion in both young, 5-month-old, and middle-aged, 12-month-old WT mice. In the current study, however, activation of orexin neurons did not have an effect on locomotion in A53T mice. As a potential explanation, this lack of effect on physical activity by orexin neuron stimulation in A53T mice may be due to existing overactivation of orexin circuitry and/or function of the mCtx, such that further activation of physical activity might not be possible. Supporting these ideas are previous studies showing pathological changes in mCtx in A53T mice (Giasson et al., 2002; Liu et al., 2014). A recent study proposes that orexin enhances locomotor activity through GAD65 neurons (Kosse et al., 2017). It is possible that inhibition of orexin neurons reduces total excitatory input to GAD65 neurons which restores their function and results in reduced locomotor activity.

The current study shows divergent effects of chemogenetic stimulation of orexin neurons on locomotion and anxiety. It is important to understand that orexins are not the sole contributors to the observed behavioral changes, although orexin neurons are undoubtedly very potent modulators of anxiety-like behavior and locomotion. It is also possible that other brain structures involved in the regulation of anxiety-like behavior and locomotion may be differentially affected by PD-associated pathology. Further, as mentioned above, A53T mice are hyperactive (Graham and Sidhu, 2010) and increasing locomotion in already hyperactive mice may be hard to achieve or detect. Similarly, A53T mice have reduced anxiety-like behavior and it is unclear whether it can be reduced even further.

The current study proposes several interesting notions: Early, pre-disease onset, behavioral changes (anxiety-like behavior and locomotion) may be associated with neurotoxic, inflammatory, and astrogliosis in Hipp and mCtx. Despite early, possibly neurotoxic, α-syn accumulations, there is no neurodegeneration of orexin neurons present in 5-month A53T mice and orexin circuitry retains neuromodulation capacity. Chemogenetic manipulation of orexin neurons can change both anxiety-like behavior and locomotion in A53T mice, suggesting that other processes regulated by orexin neurons can be modulated as well. These findings suggest a complex role of orexin neurons in this PD model and identify orexin as a potential modulator of PD-associated mood and behavioral disorders.

## Data Availability

Datasets are available on request: the raw data supporting the conclusions of this manuscript will be made available by the authors, without undue reservation, to any qualified researcher.

## Ethics Statement

All experimental procedures in this study were approved by the University of Minnesota Institutional Animal Care and Use Committee (IACUC).

## Author Contributions

MS and CMK conceived and designed the research, and interpreted the results of experiments. MS and JP performed the experiments, analyzed the data, and prepared the figures. MS drafted the manuscript. CMK edited and revised the manuscript, and approved the final version of the manuscript.

## Conflict of Interest Statement

The authors declare that the research was conducted in the absence of any commercial or financial relationships that could be construed as a potential conflict of interest.
